# Defining the Medical Profession in Mid-Nineteenth Century Bristol

**Published:** 1981

**Authors:** P. S. Brown

**Affiliations:** Department of Pharmacology, University of Bristol


### Defining the Medical Profession in Mid-Nineteenth Century Bristol

**P. S. Brown**
Department of Pharmacology, University of Bristol

The movement for reform of medical practice which occupied the middle decades of the nineteenth century culminated in the Medical Registration Act of 1858. Its provisions were a disappointment to many reformers because, among other things, it did not allow automatic prosecution of unqualified practitioners. They could only be penalised if they assumed a medical title or passed themselves off as registered practitioners. Newman[1] comments that 'the Act turned out as well as it did because it did so little' but, at least, initiation of the Medical Register seems to mark an important stage in defining who were to be, and who were not to be, considered as members of the medical profession. Doctors saw such definition as urgent, particularly at the interface between the formally qualified and those practitioners sanctioned only by tradition. It is therefore worth asking how effective the act was in achieving it. Some evidence may be provided by studying medical practitioners in a defined area before and after 1858. The providers of medical treatment in the Bristol and Clifton area in 1851 have already been surveyed, using the enumerators' books of the census and the corresponding Bristol directories.[2] To provide comparable information on medical practice after 1858, similar methods have now been applied to the directories and enumerators' books for 1861.

### UNQUALIFIED 'SURGEONS'

The most obvious group for study is that made up of individuals without formal qualifications but calling themselves 'surgeons', the description adopted by the majority of general practitioners qualified MRCS or LSA, or both. Twenty-three unqualified surgeons were identified in 1851, as against seventy-three regularly qualified general practitioners. Census returns show seven of the twenty-three without qualifications as being over 57 years old and therefore probably entitled to practise because the Apothecaries' Act of 1815 legalised unlicensed persons already practising before that date. Two others implied by their entries in directories that they were in this category, but in one case the claim is open to doubt. The surgeon concerned was Joseph Furze and he is discussed individually below, as also are others who illustrate the careers open to unqualified practitioners.

Of the remaining fourteen unqualified surgeons of 1851, eight were only found in the census returns or very briefly in the directories as well. They would form a most interesting group but little is known of them apart from what appears in the enumerators' books. Excluding them leaves six individuals who had more established practices and could still be identified in 1861. By that time four had qualified MRCS, leaving only two still unqualified: these were James Butcher and James E. Leaker who are discussed below.

By 1861, more than two years after the registration act, and despite delays in its implementation, this category of practitioner might be expected to have disappeared. This was not the case though their number had more than halved while that of the corresponding qualified practitioners had increased slightly. Ten individuals were recorded as practitioners in the directories or enumerators' book but had no traceable qualifications. Half of these, however, were only found in the single census return and in no other source: and only one of these was the head of a household, the other four being recorded as visitors or lodgers. Another, found briefly in the directories, was simply recorded as J. Smith and it is difficult on this rather general description to be certain that he was not formally qualified. And one other was a surgeon already accepted as being in practise in 1815. This leaves only three – Furze and Leaker as already mentioned, and a practitioner of similar background named Christopher Liddon.

### INDIVIDUAL PRACTITIONERS

By 1861, James Butcher, the unqualified surgeon of 1851, was no longer calling himself a surgeon and he illustrates some of the possibilities open to the unqualified. From 1848 he was described in the directories as a surgeon and chemist but was probably active earlier because 'Mr. Butcher, who practised as a surgeon on Lawrence Hill' appeared in the coroner's court in 1844. His patient had died but not before William Bird Herapath, MRCS and LSA (and later MD, FRS and FRSE), had been called in and rumours of mis-treatment by Butcher had circulated. The coroner reminded the jury that whether the medical attendant was or was not regularly licensed was immaterial to the case. They returned a verdict of death from acute bronchitis rather than the possible one of manslaughter.[3] In the 1861 census, Butcher was recorded only as a chemist, galvanist and dentist. He had begun the vigorous advertisement of a proprietary ointment and called his shop in St. James Churchyard 'Butcher's Genuine Drug and Chemical Establishment' or Phoenix House, referring to the sign of the Golden Mortar.[4] After 1858, although he no longer called himself a surgeon, he still had a wide range of activities. He was exploiting the expanding market for patent medicines and practising dentistry, which was not legally controlled, as well as medical galvanism which was probably accepted as not being medical practice under the terms of the act, a view accepted in court shortly afterwards.[5]

Joseph Furze also had a brush with the coroner when, in 1849, one of his patients died but not before relatives had called in William Bird Herapath. The coroner said Furze represented himself as a surgeon but appeared in the directories as a druggist. Again the jury was told the formal qualifications were immaterial and returned a verdict of death from natural causes.[6] Furze claimed legality by being in practice before 1815. This is possible as, according to census returns, he would have been 19 at the time: but he did not appear on the Medical Register after the act, suggesting that he could not prove his point. Christopher Liddon also claimed to have been in practice before 1815 but census returns show that he would have been only 15 and, not surprisingly, he also did not appear on the register. In the 1840s he had been a chemist and dentist and then switched to being a surgeon and chemist. In the 1861 census he appeared only as a chemist, but in the directories was still described as a surgeon as well.

The last of those for individual discussion is James Leaker who had a well-established practice as a surgeon in Castle Street and later in Old Market. He appeared before the magistrates in 1860 for contravening the Medical Act, on information laid by the physician, J. P. Macdonald, secretary of the Bristol and Bath Medical Registration Association. Though unqualified, Leaker described himself as a surgeon on a notice in his window and elsewhere; but surprisingly the case was dismissed as the magistrates thought he had not used the title 'willfully and falsely'. They may have been unwilling to deal harshly with a well-established practitioner though his practice might now be illegal. Even the registration association appears to have been slow and reluctant to act. A year previously they had simply asked Leaker to remove the sign from his window: this he did, but he later replaced it.[7]

### LEGAL CONSTRAINTS BEFORE 1858

The medical Registration Association for Bristol, Bath and neighbourhood was founded late in 1859 and seems to have been preceded by a Bristol association.[8] Before 1858, the Apothecaries' Act of 1815 was occasionally used as a legal weapon against the unqualified. At Bristol assizes in 1846, the Company successfully prosecuted a person called Wall for practicising as an apothecary without their license.[9] The regular practitioners also tried to exert pressure through coroners' courts where they held a strong position, being called on to establish the cause of death. But, as already illustrated, formal medical qualification was not an issue in these courts.

In both the inquests mentioned, William Bird Herapath was involved. This may be a coincidence or it may be that Herapath was, for one reason or another, particularly anxious to expose the 'quacks'. His interest in forensic chemistry may have made him alert to the possibilities of therapeutic misadvanture and, practising from Old Market, he was close to areas rich in unqualified practitioners.[2] The medical profession, particulaly when urging medical reform, had to be seen to be above reproach and this is illustrated by an incident involving the Herapaths. A Mr. W. Herapath, presumably the surgeon's father who was a respected citizen and a distinguished toxicological chemist, was required at Bristol Quarter Sessions to pay nominal damages for kissing the attractive 20-year old wife of James Wildgoose, a brick and tile maker. This resulted in a ballad on the subject being sung in the Bristol streets, and the rapid appearance of a note in the newspapers that the Herapath concerned was not the medical member of the family.[10]

### 'CONFIDENTIAL MEDICAL ADVICE'

One type of irregular practice was conspicuous because it was carried out through advertisements for confidential medical advice in conditions which might range from venereal disease to impotence. The practitioners were often described as consulting surgeons or physicians, and some published booklets, designed presumably to frighten suggestible patients to consult them in the belief that they suffered from venereal infections or from what was described as the results of solitary indulgence. In the survey of 1851, three such businesses were found by their newspaper advertisements: two of the individuals involved were also identified in the enumerators' books where both prudently described themselves only as medicine vendors.[2]

A sample of Bristol newspapers during the next decade yielded four frequent advertisers apparently of this type: no doubt there were others not included in the sample. Dr. Robert Woodman ran a Private Medical Establishment for diseases of a delicate nature; Messrs. W. Anderson & Co. were old-established surgeons offering private medical advice 'to the Gay and Indiscreet'; Messrs. Henry & Co., surgeons, supplied similar advice and sold Purifying Botanic Pills; and Dr. J. A. Blake of Queen's Square supplied a booklet on nervousness, debility and general decay of the faculties, and sold Peruvian Drops in the form of lozenges which effectively eradicated all diseases of the generative organs in three days.[11] Both Anderson and Henry claimed that they held the Apothecaries' license, but usually it is difficult to locate the individuals behind the advertisements and highly likely that many were not medically qualified. Some firms may have taken the precaution of recruiting a qualified partner after 1858, as is suggested by an advertisement quoted in the *Lancet* for a medically qualified man to join an 'Advertising Medical Consulting Practice'.[12]

The advertisements of this type of practitioner roused indignation both by the indelicacy of the matters discussed, even if only obliquely, and by the style of practice they reflected. They even stimulated the formation of a Union for the Discouragement of Vicious Advertisements which tried to dissuade newspaper editors from accepting this material.[13] *Punch* developed the theme and was quoted with approval by a Bristol newspaper which, naturally, proclaimed itself to be above reproach.[14] But newspapers were not the only vehicle of advertisement and it was said that Dr. Blake had 'touters who distribute small offensive bills – to the great annoyance of the public' and that he was popularly known as the author of the 'Bristol Bridge nuisance'.[15] And advertisements did not cease after 1858, Anderson & Co. still advertising regularly in the *Bristol Mercury* during the early months of 1859. Blake also continued to advertise and became a target for the Medical Registration Association.

The person behind Blake & Co. of Queen's Square turned out to be Isaac Jacques who was summoned on information laid by Dr. Macdonald. A young ship's mate had read Blake's publication entitled *The Secret Preceptor* which alarmed him into seeking advice at the author's address where Jacques presented himself as 'the doctor', diagnosed a serious case without examining the patient, supplied medicines and extracted payment. Apparently the case for the Registration Association was not sufficiently supported in some respect and the charge was dismissed, though Jacques was clearly not medically qualified and seems to have been taking the title of doctor.[16]

Even if unsuccessful, legal actions must have persuaded practitioners of this type to alter their strategy. Certainly no offers by Bristol practitioners of confidential medical advice framed in the characteristic manner were found in the *Bristol Mercury* for 1861. One possible change of tactics was discussed by a Bristol surgeon writing to the *Lancet.* He suggested that all an irregular practitioner need do was to pay the patent medicine tax and see that all his preparations carried the government stamp: he would then be safe from the law.[17]

### PRESCRIBING DRUGGISTS AND OTHERS

Individuals practicing as chemists and unqualified surgeons who were identified in the census returns have already been discussed. A further eleven persons described both as surgeons and as chemists or druggists were found in newspapers or directories between 1841 and 1861. Three had regular medical qualifications and were legitimately combining the two activities. This was not considered unusual in the 1840s as was shown by an advertisement in a newspaper for a good retail drug business in Bristol, stating that 'a surgeon would find it an excellent opportunity'.[18] Persons combining both activities were less common in the 1850s, after 1858 none was found other than those already discussed. Many irregular practitioners may simply have called themselves druggists, and there seems little doubt that a great deal of 'counter practice' was done by chemists and druggists in Bristol and elsewhere.[2]

There were numerous other groups offering medical treatment, including mesmerists, medical galvanists and herbalists. Scraps of evidence suggest also that a great deal of informal healing was practiced, for example by the stone mason 'who had acquired a reputation for curing all sorts of diseases',[19] or treatment for enlarged glands in the neck by a woman from Lawrence Hill 'who professed to be skilful in such cases'.[20] Most of these practitioners do not concern us as they did not assume a medical title, though a few did. Among them were the American herb doctors who are discussed elsewhere;[21] but it is worth recording here one success for the Medical Registration Association. This was in the case of Thomas Airey, a herb doctor whose flamboyant advertisements described him as MRCS and MRCP.[22] His unsuccessful defence in court was that these memberships were of the Reformed Colleges of Surgeons and Physicians, respectively, of New York.[23]

### CONCLUSIONS

The Medical Registration Act did not accomplish the sudden disappearance of unqualified practitioners. Medical practice had embraced such a wide range of dissimilar activities that no legislation could have produced instant conformity. And the act did not change the social conditions that had called for a variety of styles of practice. The same patient may have used different types of practitioners for different purposes. The case is recorded of a Bristol woman whose delivery was supervised by a regular practitioner but who then called in a herb doctor to treat the painful swollen leg that followed.[24]

Some unqualified practitioners may have changed their designation as a result of the act, and James Butcher illustrates this side-stepping out of trouble. Some may have put the government stamp on their wares, but the figures (quoted by Chapman[25]) do not show any dramatic jump in receipts from the medicine tax around 1858. But many of the irregular practitioners seem to have been little troubled by the Medical Act and a Royal Commission, reporting in 1882, quoted evidence for the continuation of unqualified practice.[26] The report admitted that the act of 1858 had failed to deal with the problem and this view accords with the early findings from Bristol. This is, however, to focus on a negative aspect of the act and its significance was not that it failed to penalise a small section of outsiders, but rather that the inception of the General Council and the Medical Register was an important step towards defining and formalising the boundaries of the medical profession.

### REFERENCES

1 NEWMAN, C., The evolution of medical education in the nineteenth century, London, Oxford University Press, 1957, p.192.

2 BROWN, P. S., The providers of medical treatment in mid-nineteenth century Bristol, *Medical History,* 1980, **24**, 297.

3 *Bristol Mirror,* 28 December 1844.

4 *Bristol Mercury,* 23 January and 8 May 1858.

5 *Times,* 11 June 1861, p.11b.

6 *Bristol Times,* 7 July 1849.

7 *Bristol Mercury,* 24 November 1860.

8 *Bristol Mirror,* 13 August and 12 November 1859.

9 *Bristol Gazette,* 20 August 1846.

10 *Bristol Mirror,* 2 and 9 July 1853.

11 *Bristol Mercury,* 15 January 1859.

12 Quackery on its deathbed, *Lancet,* 1858, 2, 405.

13 *Lancet,* 1851, **1**, 72.

14 Poisonous puffs, *Bristol Mirror,* 16 July 1853.

15 *Bristol Observer,* 14 July 1860.

16 *ibid.,* 7 July 1860.

17 The Medical Reform Bill and quakery, *Lancet,* 1854, **1**, 218.

18 *Bristol Mercury,* 20 August 1842.

19 *Bristol Gazette,* 23 March 1843.

20 *Bristol Mirror,* 20 April 1850.

21 BROWN, P. S., Herbalists and medical botanists in mid-nineteenth century Britain, with special reference to Bristol, *in preparation.*

22 *Bristol Mercury,* 5 November 1859.

23 *Bristol Mirror,* 12 and 19 November 1859.

24 *Bristol Mercury,* 6 and 13 November 1852.

25 CHAPMAN, S., Jesse Boot of Boots the Chemists, London, Hodder & Stoughton, 1974, p.203.

26 Report of the Royal Commission on the Medical Acts, 1882 (C3259), p.xiii, pp.247-250.

